# Signatures of selection and drivers for novel mutation on transmission-blocking vaccine candidate *Pfs25* gene in western Kenya

**DOI:** 10.1371/journal.pone.0266394

**Published:** 2022-04-07

**Authors:** Kevin O. Ochwedo, Shirley A. Onyango, Collince J. Omondi, Pauline W. Orondo, Benyl M. Ondeto, Ming-Chieh Lee, Harrysone E. Atieli, Sidney O. Ogolla, Andrew K. Githeko, Antony C. A. Otieno, Wolfgang R. Mukabana, Guiyun Yan, Daibin Zhong, James W. Kazura

**Affiliations:** 1 Department of Biology, Faculty of Science and Technology, University of Nairobi, Nairobi, Kenya; 2 Sub-Saharan Africa International Centre for Excellence in Malaria Research, Homa Bay, Kenya; 3 School of Zoological Sciences, Kenyatta University, Nairobi, Kenya; 4 Department of Biochemistry, Jomo Kenyatta University of Agriculture and Technology, Nairobi, Kenya; 5 Program in Public Health, College of Health Sciences, University of California, Irvine, Irvine, California, United States of America; 6 School of Public Health and Community Development, Maseno University, Kisumu, Kenya; 7 Centre for Global Health Research, Kenya Medical Research Institute, Kisumu, Kenya; 8 Centre for Global Health and Diseases, Case Western Reserve University, Cleveland, Ohio, United States of America; Ehime Daigaku, JAPAN

## Abstract

**Background:**

Leading transmission-blocking vaccine candidates such as *Plasmodium falciparum* surface protein 25 (*Pfs25* gene) may undergo antigenic alterations which may render them ineffective or allele-specific. This study examines the level of genetic diversity, signature of selection and drivers of *Pfs25* polymorphisms of parasites population in regions of western Kenya with varying malaria transmission intensities.

**Methods:**

Dry blood spots (DBS) were collected in 2018 and 2019 from febrile outpatients with malaria at health facilities in malaria-endemic areas of Homa Bay, Kisumu (Chulaimbo) and the epidemic-prone highland area of Kisii. Parasites DNA were extracted from DBS using Chelex method. Species identification was performed using real-time PCR. The 460 base pairs (domains 1–4) of the *Pfs25* were amplified and sequenced for a total of 180 *P*. *falciparum*-infected blood samples.

**Results:**

Nine of ten polymorphic sites were identified for the first time. Overall, *Pfs25* exhibited low nucleotide diversity (0.04×10^−2^) and low mutation frequencies (1.3% to 7.7%). Chulaimbo had the highest frequency (15.4%) of mutated sites followed by Kisii (6.7%) and Homa Bay (5.1%). Neutrality tests of *Pfs25* variations showed significant negative values of Tajima’s *D* (-2.15, p<0.01) and Fu’s F (-10.91, p<0.001) statistics tests. Three loci pairs (123, 372), (364, 428) and (390, 394) were detected to be under linkage disequilibrium and none had history of recombination. These results suggested that purifying selection and inbreeding might be the drivers of the observed variation in *Pfs25*.

**Conclusion:**

Given the low level of nucleotide diversity, it is unlikely that a Pfs25 antigen-based vaccine would be affected by antigenic variations. However, continued monitoring of Pfs25 immunogenic domain 3 for possible variants that might impact vaccine antibody binding is warranted.

## Introduction

Over the past decade remarkable progress has been made in reducing the global malaria burden by coordinated public health interventions targeting both vectors and parasites [1]. However, progress in malaria control has been stalled partly due to the spread of insecticide and antimalarial drug resistance [1, 2]. One of the interventions needed to reduce the malaria burden is an effective *P*. *falciparum* vaccine [3–5]. Barriers to advances in vaccine development include allelic variation of target antigens due to mutations in their immunodominant protein domains [6–10]. Currently, there are attempts towards developing transmission-blocking vaccines (TBVs) that target *P*. *falciparum* sexual stage parasites [5, 11]. TBVs elicit host antibody responses that block the sporogonic cycle of the parasite in the mosquito vector, thereby thus reducing and, ultimately, stopping ongoing transmission in local communities.

Among the various TBVs under development a vaccine based on the Pfs25 protein sequence is perhaps the most advanced [12–15]. The cysteine-rich 25 kilo-Dalton molecule is expressed in the protease-rich mosquito vector midgut post-fertilization, where it facilitates ookinete epithelium penetration, aggregation, and maturation to oocysts [16, 17]. Protein expression of the Pfs25 protein is translationally repressed in the human host by the Pumilio/FBF family RNA-binding protein [18, 19]. The Pfs25 protein consists of four tandem epidermal growth factor (EGF) domains with 22 cysteine residues that is anchored on the parasite surface by a C-terminal glycosylphosphatidylinositol (GPI) [20]. Recombinant Pfs25 antigen in combination with other TBV antigens [12, 21] or alone have been shown to elicit anti-Pfs25 antibodies [13, 22–27] that block oocyst development in standard membrane feeding assays. Notably, translational repression of Pfs25 protein expression in the human host tempers enthusiasm for field deployment of a TBV based on this antigen alone due to the absence of natural boosting in malaria endemic communities [25, 27].

The *Pfs25* exon is considered to be highly conserved despite the existence of various sites with mutations [28–30]. Documented mutations include locus 392 in *P*. *falciparum* isolates from China and India and locus 428 of isolates from Cambodia and India [29–31]. The aim of this study was to determine the distribution of polymorphic sites, level of genetic diversity and identify possible signatures of selection in the four domains of the Pfs25 protein from *P*. *falciparum* parasites circulating in malaria-endemic and epidemic-prone regions of western Kenya.

## Materials and methods

### Study site and sampling

**Fig 1:**
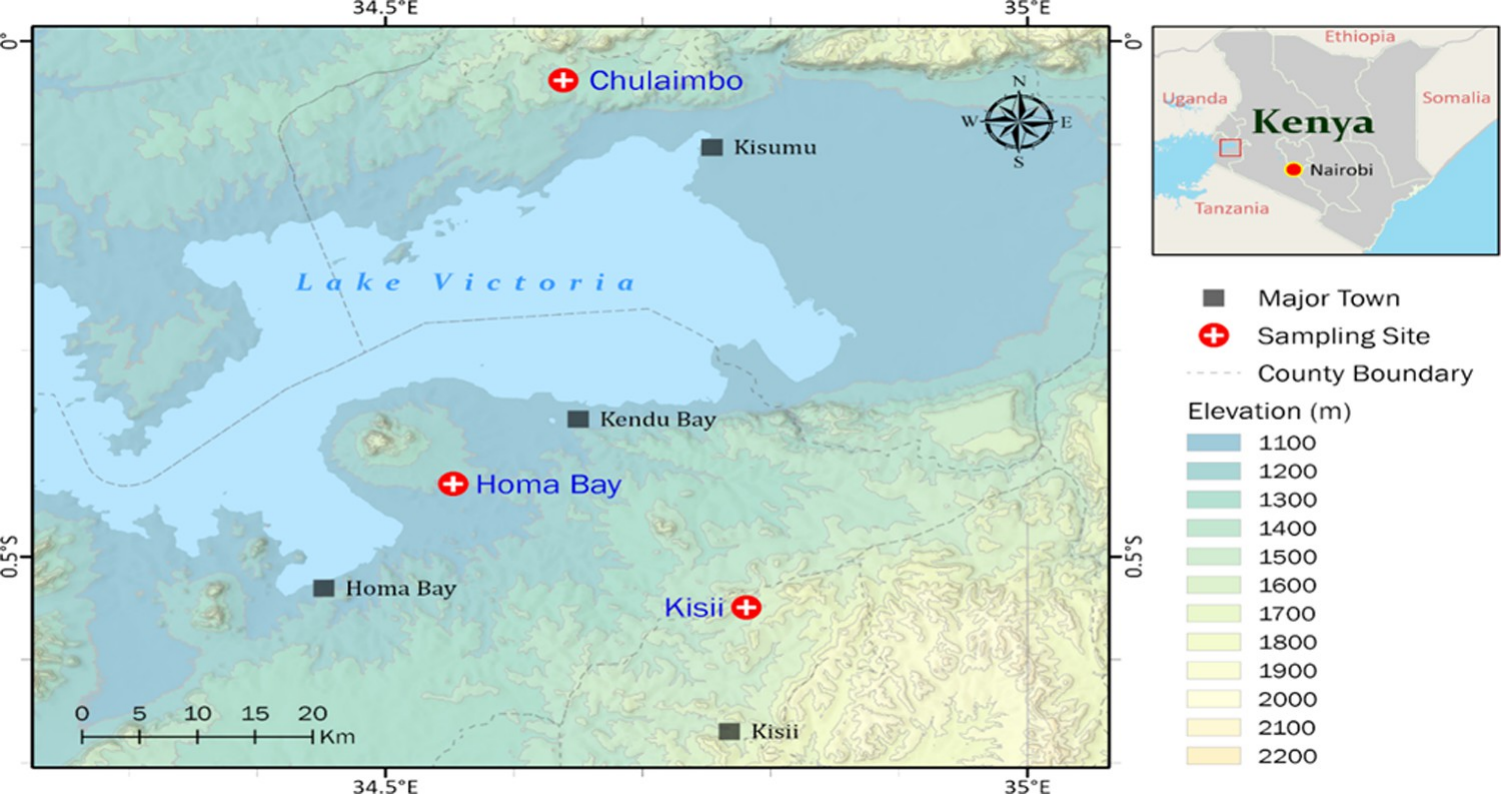
Homa Bay County, Kisumu County (Chulaimbo) and Kisii County study sites in western Kenya. Circles with positive signs represent sampling points where dry blood spots (DBS) of patients presenting with malaria symptoms were collected. The colour changes on the land background represent variation in elevation. This figure was prepared with ESRI ArcGIS Pro 2.8 with field survey results and publicly available datasets. The map contains information from OpenStreetMap and OpenStreetMap Foundation, which is made available under the Open Database License.

A cross-sectional study was conducted in 2018 and 2019 in health clinics in malaria-endemic areas of Homa Bay and Kisumu (Chulaimbo) Counties and the malaria epidemic-prone highlands of Kisii County (**Fig 1**). A total of 302 patients presenting with fever and symptoms of uncomplicated malaria, e.g., myalgia, fatigue, non-localizing symptoms, were recruited to donate a finger prick blood sample. 138 patients were from Homa Bay County, 62 from Kisumu County (Chulaimbo) and 102 from Kisii County. Health facilities selected for sample collection in Homa Bay lie at 34.64190°E-0.38000°S and 1134–1330 metres above the sea level (asl), in Kisumu (Chulaimbo Sub County hospital) at 00.03572°S-034.62196°E and 1328–1458 m asl and in Kisii (Eramba health dispensary) at 34°48′E, 00°35′S; 1540–1740 m asl (**Fig 1**).

Four samples of 25 μL of blood were collected on coded Whatman™ Blood Stain Cards (GE Healthcare WB100014) containing details such as the patient’s study code, date of collection, age, and gender as previously described [32]. Each of the collected coded Whatman™ Blood-Stained Card was singly placed in individual plastic bags containing silica gel before being enclosed together in a labelled envelope and transported to the International Centre of Excellence for Malaria Research (ICEMR) laboratory located at Tom Mboya University College in Homa Bay town for storage at -20˚C and later molecular processing.

### DNA extraction

Genomic DNA was extracted from the 302 DBS samples using the Chelex resin (Chelex -100) method following the published method [33] with modifications. Briefly, a 3mm ring of blotted filter paper containing a blood sample was punched into a sterile coded 1.5ml Eppendorf tube using a sterile craft store puncher. 950μl of 1×PBS and 50μl of 10% saponin was added and mixed well before incubation at 4°C overnight. The mixture was centrifuged at 12,000 rpm for 10 min at room temperature before discarding the supernatant content. Any remnant of saponin was washed by adding 1ml PBS and spinning at 12,000 rpm for 5 min. The PBS was discarded and the tube content spun for 15sec before removal of the liquid component using the P200 pipette. The Eppendorf tubes containing filter paper were air-dried at room temperature for 15min. 250μl (20%) of Chelex suspension was added and the mixture was incubated in a water bath maintained at 85°C for 10min. During incubation, the mixture containing DNA was vortexed for 5min to suspend the extracted DNA. Upon completion, the mixture was spun at 12,000 rpm for 1 min and the DNA was transferred into a coded clean 0.5ml Eppendorf tube and stored at -20°C. Cultured *P*. *falciparum* laboratory strain NF54 genomic DNA was also extracted and used as a positive control for PCR and sequencing.

### Detection of *Plasmodium falciparum* infections

Real-time (RT-PCR) with species-specific 18s rRNA and probes were used to identify *P*. *falciparum* infections as previously described [34]. Briefly, RT-PCR was set with a final volume of 12μl containing 2μl of sample DNA, 6μl of PerfeCTa® qPCR ToughMix™, Low ROX™ Master mix (2X), 0.5μl of the species-specific probe, 0.4μl of the species-specific forward primers (10μM), 0.4μl of the species-specific reverse primers (10μM) and 0.1μl of double-distilled water. The thermal profile used was set at 50°C for 2 min, followed by 45 cycles of (95°C for 2 min, 95°C for 3 sec and 58°C for 30 sec). Three positive samples from laboratory strain and three negative samples from blank filter paper were used as a positive and negative control for RT-PCR respectively. The RT-PCR amplification was performed on QuantStudio 3 Real-Time PCR System (ThermoFisher, Carlsbad, CA, USA). A total of 180 samples were confirmed positive for *P*. *falciparum* by RT-PCR. The level of parasitemia ranged from 120 to 146,880 parasites/μL blood with a median of 4,920 parasites/μL.

### *Pfs25* gene amplification and sequencing

A total of 180 *P*. *falciparum* infected samples (78 from Homa Bay, 40 from Chulaimbo, and 62 from Kisii) confirmed positive by RT-PCR were used to amplify the target Pfs25 gene. Amplification of the 460bp exon was performed through nested PCR in T100™ Thermal Cycler (Bio-Rad) as previously described [35]. Briefly, for Nest I PCR, a final reaction volume of 13μL was prepared by addition of 3μL of parasite DNA to a mixture containing 5.75μL of DreamTaq Green PCR Master Mix (2X), 0.5μL of Nest I forward primer (10μM), 0.5μL of Nest I reverse primer (10μM) and 3.25μL of double-distilled water. For Nest II, a final reaction volume of 23μL was prepared by addition of 3μL of Nest I amplicon to a mixture containing 11.5μl of DreamTaq Green PCR Master Mix (2X), 0.5μL of Nest I forward primer (10μM), 0.5μL of Nest I reverse primer (10μM) and 7.5μL of double-distilled water. Nested I and II PCR conditions were set as follows: initial denaturation at 94°C for 3 minutes, 34 cycles of denaturation at 94°C for 30 sec, annealing at 50°C for 30 sec, primer extension at 68°C for 1 min and final extension at 72°C for 6 min. PCR amplification was performed on T100™ Thermal Cycler (Bio-Rad, Hercules, CA, USA). The quality of the 460bp amplicons from Nested II PCR were assessed by performing gel electrophoresis in 1.5% w/v agarose gel after amplification and before sequencing. Each of the 180 PCR products was purified by the addition of Exonuclease I and Shrimp Alkaline Phosphatase (ExoSAP-IT) and incubated at 37°C for 15 min. The ExoSAP-IT in purified PCR products was inactivated by heating at 80°C for 15 min. The cleaned PCR products with quantity above 25ng/μl were selected for sequencing after testing 1μl of each using NanoDrop™ Lite Spectrophotometer (Thermo Scientific™). Bi-directionally sequencing was done using BigDye^®^ Terminator v3.1 Sequencing Standard kit on ABI PRISM^®^ 3700 DNA Analyzer (Applied Biosystems, Foster City, CA, USA). Repeat sequencing was done on samples with poor reads to confirm mutated sites.

### Ethics approval

The study was approved by the Maseno University Ethics Review Committee (MUERC protocol No. 00456) and the University of California, Irvine Institutional Review Board (HS#2017–3512) and received authorization from the Ministry of Health. All volunteers or their guardians gave written informed consent to participate in providing blood samples.

### Data analysis

The sequence assembly was done using Geneious version 11.1.5 software. Multiple sequence alignment was done using ClustalW, a matrix-based algorithm that is in build in Mega X software [36]. Segregating sites, mutated codons, and type of mutation were determined by inputting sequences from each region and reference sequence NF54 (Accession number X07802.1) in CodonCode Aligner version 9.0.1 (CodonCode Corporation, www.codoncode.com). Mixed haplotype infections were defined as that sample had sequencing chromatogram with double peaks (second peak higher that 50% of first peak). The PHASE function of DnaSP Version 6.12.03 was used to infer the haplotypes of mixed infections. The Pfs25 antigen structural delineation was done by inputting full-length sequence (X07802.1) on Protein Homology/analogY Recognition Engine (PHYRE2) version 2.0. The generated Pfs25 model by Phyre2 was visualized, modified and mutated sites marked in UCSF Chimera version 1.15 [37]. The genetic diversity indices; nucleotide diversity (π) [38], the mean number of pairwise difference (k) [39], number of segregating sites (S), number of haplotypes (h), haplotype diversity (*H*d) were computed per region from the aligned sequences using DnaSP software [40] and results confirmed using Arlequin version 3.5.2 [41]. Haplotype data generated from DnaSP in nexus format with slight modification was used to draw a haplotype network using Population Analysis with Reticulate Trees (Popart) version 1.7 software [42]. The ratio of nonsynonymous to synonymous mutations (d_N_/d_S_) was computed to give a hint on selection pressure acting on Pfs25 antigen from this region [43–46]. Presence and type of natural selection or deviation from the standard neutral model were determined through computation of neutrality tests such Tajima’s *D* [47], Fu and Li’s *D*, Fu and Li’s *F* and Fu’s *Fs* statistics [48]. The output of Tajima’s *D* values were exported to GraphPad version 8.3.0 and used to generate the Tajima’s *D* curve for both regions. The negative values from these tests correspond to a purifying natural selection whereas positive values are a representation of positive selection. Other factors or drivers sustaining the observed mutations such as recombination and inbreeding were confirmed by detecting the presence or absence of Recombination events (Rm) and Linkage disequilibrium (LD) were confirmed using DnaSP software.

## Results

### Distribution of polymorphisms in four domains of Pfs25 antigen

A total of 10 segregating or polymorphic sites (123, 124, 249, 330, 345, 364, 372, 390, 394 and 428) were identified across the 460bp of *Pfs25* gene from *P*. *falciparum* isolates that were in circulation in western Kenya (**S1 Fig**). Homa Bay had 3/78 (3.8%) mutated sequences, Chulaimbo had 3/39 (7.7%), and Kisii had 3/60 (5%). The polymorphic sites were observed at a frequency of 7.9% (14/177) among sampled parasites (**Table 1**). The distribution of these polymorphic sites per study area was as follows: Homa Bay 5.1% (4/78), Chulaimbo 15.4% (6/39) and Kisii 6.7% (4/60). Most (80%) of the polymorphic sites (loci 123, 124, 249, 330, 345, 364, 372 and 428) were singletons or single nucleotide polymorphs (SNPs) whereas others (loci 390 and 394) were parsimony informative as they were observed in more than one sample. Three samples showed mixed infections of mutant/wild type (**S1 Fig, S1 File**). Transition and transversion equally contributed to the observed mutations across the 10 segregating sites (**Table 1**). The base substitution resulted in more (60%) synonymous amino acid or codon changes compared to nonsynonymous. Nonsynonymous changes in *Pfs25* sequences from Homa Bay resulted in codon change L42M (Leucine to Methionine), whereas synonymous changes included H41H (Histidine to Histidine), C110C (Cysteine to Cysteine), and T142T (Threonine to Threonine). Each of these changes occurred at a 1.3% frequency, with two (H41H and T124T) occurring on the same sequence. On polymorphic sequences in Chulaimbo, synonymous T130T (Threonine to Threonine) and nonsynonymous mutation V132I (Valine to Isoleucine) occurred as a pair at a frequency of 7.7% each. Kisii parasites had codon changes I83I (Isoleucine to Isoleucine), C115W (Cysteine to Tryptophan), L122L (Leucine to Leucine), and V143G (Valine to Glycine), which were observed at a frequency of 1.61% each (**Table 1**). Polymorphic codons L122L and V143G occurred on a single sequence.

**Table 1:** Polymorphic sites in Pfs25 sequences from Homa Bay, Kisumu (Chulaimbo) and Kisii region in western Kenya.

Segregating sites	Domain	Allelic frequency			Substituted bases	Type of Substitution	Codon change	Type of mutation
		Homa Bay n (%)	Chulaimbo n (%)	Kisii n (%)				
123	D1	1 (1.3)	-	-	T/C	Transition	H41H	Syn
124	D1	1 (1.3)	-	-	T/A	Transversion	L42M	Nsyn
249	D2	-	-	1(1.7)	A/T	Transversion	I83I	Syn
330	D3	1 (1.3)	-	-	T/C	Transition	C110C	Syn
345	D3	-	-	1(1.7)	T/G	Transversion	C115W	Nsyn
364	D3			1(1.7)	T/C	Transition	L122L	Syn
372	D3	1 (1.3)			A/G	Transition	T124T	Syn
390	D3	-	3 (7.7)	-	T/A	Transversion	T130T	Syn
394	D3	-	3 (7.7)	-	G/A	Transition	V132I	Nsyn
428	D3	-	-	1(1.7)	T/G	Transversion	V143G	Nsyn

n represents the proportion of sequences per study site with polymorphic sites.

**Fig 2:**
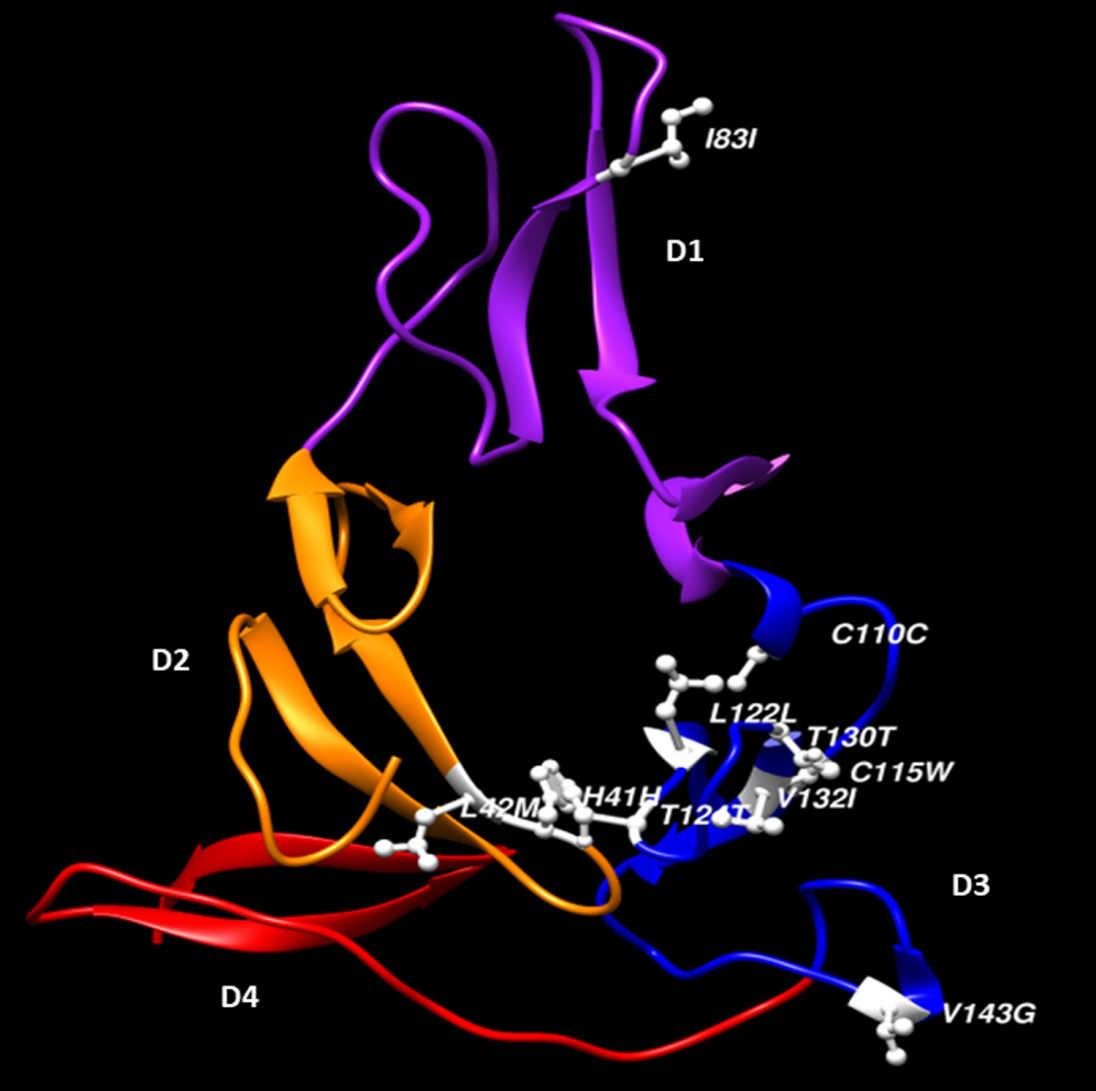
Predicted structure of Pfs25 antigen and distribution of polymorphic codons on its domain 1, 2 and 3. Beta strands (β) and loops in orange represent D1; D2 is in purple β-strands and loops; D3 is in blue β-strands and loops whereas D4 is represented by red β-strands and loops. The white balls and sticks represent codons with mutations in the Pfs25 antigen (Molecular graphics and analyses performed with UCSF Chimera, developed by the Resource for Biocomputing, Visualization, and Informatics at the University of California, San Francisco, with support from NIH P41-GM103311).

Out of the mutated 10 codons in Pfs25 antigen, 7 (330, 345, 364, 372, 390, 394 and 428) were present in D3 (**Fig 2**). Domain 2 had 1 (I83) mutated codon whereas D1 had two mutated codons (H41 and L42) which were close to D3 codons. In D3, mutated codon C110, L122, T130 and V132 were within structural proximity.

### Genetic diversity indices of *Pfs25* gene

**Fig 3:**
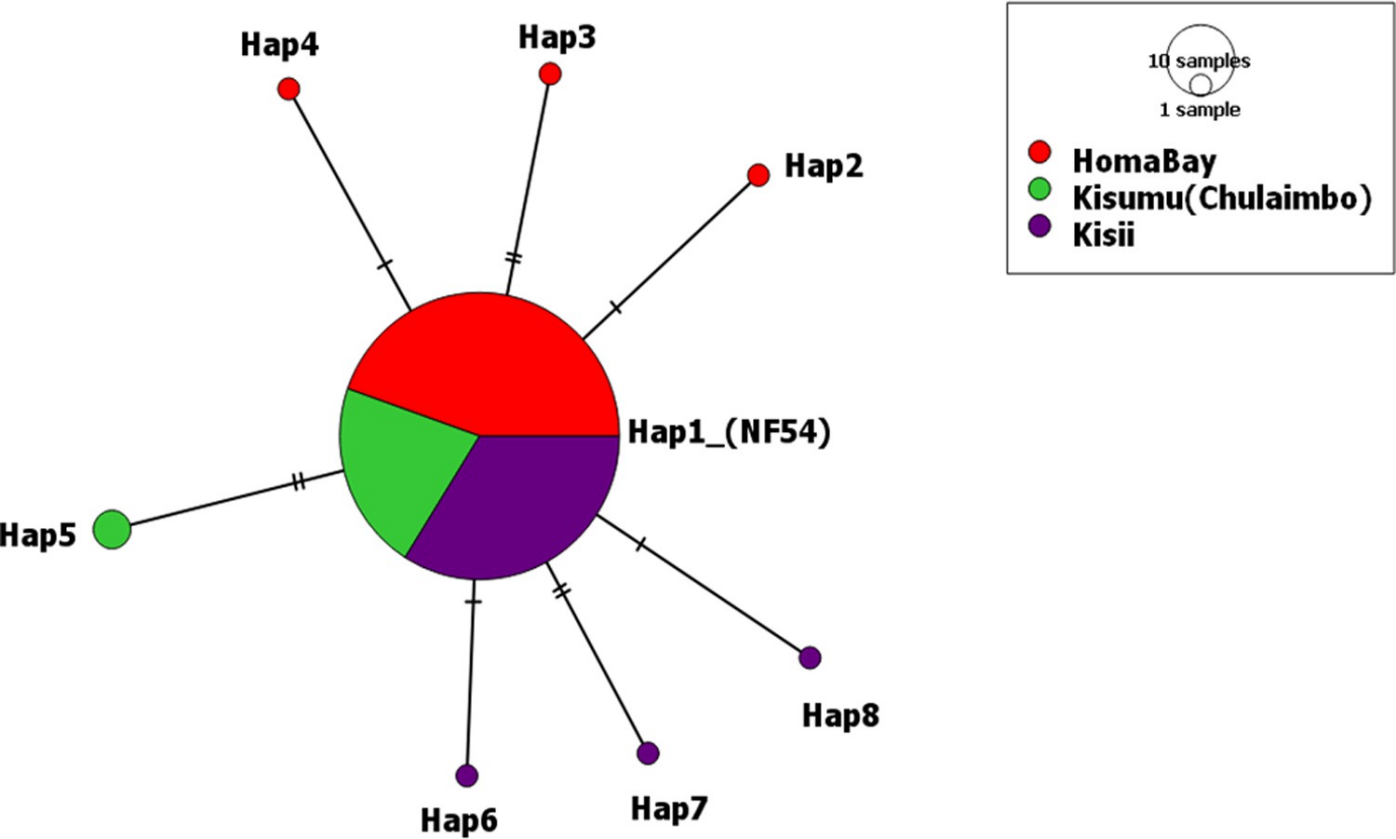
TCS-network analysis of the relationship of Pfs25 haplotypes in malaria-endemic and epidemic-prone regions of western Kenya. The network shows the distribution of haplotype within malaria-endemic lowlands of Homa Bay (red) and Kisumu (Chulaimbo) (green) as well as Kisii highlands (purple). The hatch marks represent the number of mutations (double hatch marks equate to two polymorphic sites) whereas the size of the circle equate to the relative frequency of haplotypes. Hap 1 (lacked mutated site, identical with Pfs25 reference sequence of NF54 strain or 3D7 strain), Hap2 (had codon change L42M), Hap3 (H41H and T124T), Hap4 (C110C), Hap5 (T130T and V132I), Hap6 (I83I), Hap7 (C115W) and Hap8 (L122L, V143G).

Relatively low nucleotide diversity (π) was observed among the sequences analysed. The 177 Pfs25 sequences had π of 0.04×10^−2^, k of 0.16 and a nucleotide conservation index of 97.7% that slightly varied per study site (**Table 2**). Chulaimbo isolates had the highest observed π (0.07×10^−2^) and Hd (0.15) followed by Kisii (π = 0.03 and Hd = 0.10) then Homa Bay (π = 0.02 and Hd = 0.08). There was no significant difference (p = 0.400) when the population pairwise FSTs were computed for Homa Bay and Kisii sequences (FST = 0.00). Similar results (p = 0.160) was observed for population differences between Chulaimbo and Kisii (FST = 0.037). No significant difference (p = 0.110) in observed allelic variation between Homa Bay and Chulaimbo sequences (FST = 0.039). The highest diversity index in Chulaimbo was due to a high frequency of T130T and V132I codon changes (**Table 3**). Homa Bay and Kisii had the highest number of Pfs25 haplotypes (four each) compared to Chulaimbo (**Fig 3**). Among analysed samples from Chulaimbo, blood samples of patients from the same household were found to harbour different haplotypes. Six blood samples, 3 from each different household had either Hap1 with no mutated sites or Hap5 with both mutated codon T130T and V132I. This was however not observed in sequences from Homa Bay and Kisii.

**Table 2:** Summary of genetic diversity indices of *Pfs25* gene in western Kenya.

Study site	N	C (%)	S	π (×10^−2^)	H	*Hd*	Ks or d_*S*_ (×10^−2^)	Ka or d_N_ (×10^−2^)	d_N_/d_S_	Tajima’s *D*	Fu’s *F*_*S*_
Homa Bay	78	99.10	4	0.02	4	0.08	0.09	0.01	12.29	-1.81*	-4.42 *
Chulaimbo	39	99.50	2	0.07	2	0.15	0.16	0.04	3.93	-0.73	0.80
Kisii	60	99.10	4	0.03	4	0.10	0.07	0.02	3.89	-1.84*	-3.90*
All sites	177	97.70	10	0.04	8	0.10	0.10	0.02	5.37	-2.15*	-10.91*

N: Sample size; C: Conservation index; S: Segregating sites; π: nucleotide diversity; H: haplotype; *Hd*: Haplotype diversity; Ks or d_*s*_: The number of synonymous (or silent) substitutions per synonymous (or silent) site by Jukes and Cantor; Ka or d_*N*_: The number of nonsynonymous substitutions per nonsynonymous site by Jukes and Cantor; d_N_/d_S_: Ratio of nonsynonymous and synonymous substitutions

*: Significance (p<0.05).

**Table 3:** Pfs25 haplotypes frequencies across Homa Bay, Chulaimbo and Kisii regions.

Distribution of Pfs25 haplotypes					
Homa Bay					
Haplotype	Codon Change	Number of Sequences	Haplotype Frequencies (%)	LD χ ^2^ (Loci)	r^2^
Hap1	0	75	96.15	79.00^***B^ (Locus 123 and 372)	0.02×10^−2^
Hap2	L42M	1	1.28		
Hap3	H41H, T124T	1	1.28		
Hap4	C110C	1	1.28		
**Chulaimbo**					
Hap1	0	36	92.31	40.00^***B^ (Locus 390, 394)	1
Hap5	T130T, V132I	3	7.69		
**Kisii**					
Hap1	0	57	95.00	61.00^***B^ (Locus 364 and 428)	1
Hap6	I83I	1	1.67		
Hap7	C115W	1	1.67		
Hap8	L122L, V143G	1	1.67		

LD: Linkage disequilibrium; **χ** ^**2**^: chi-square test; ^*B^: Significant by the Bonferroni procedure; Rm: Minimum number of recombination events.

### Signature of selection and other mutation drivers on *Pfs25* gene

**Fig 4:**
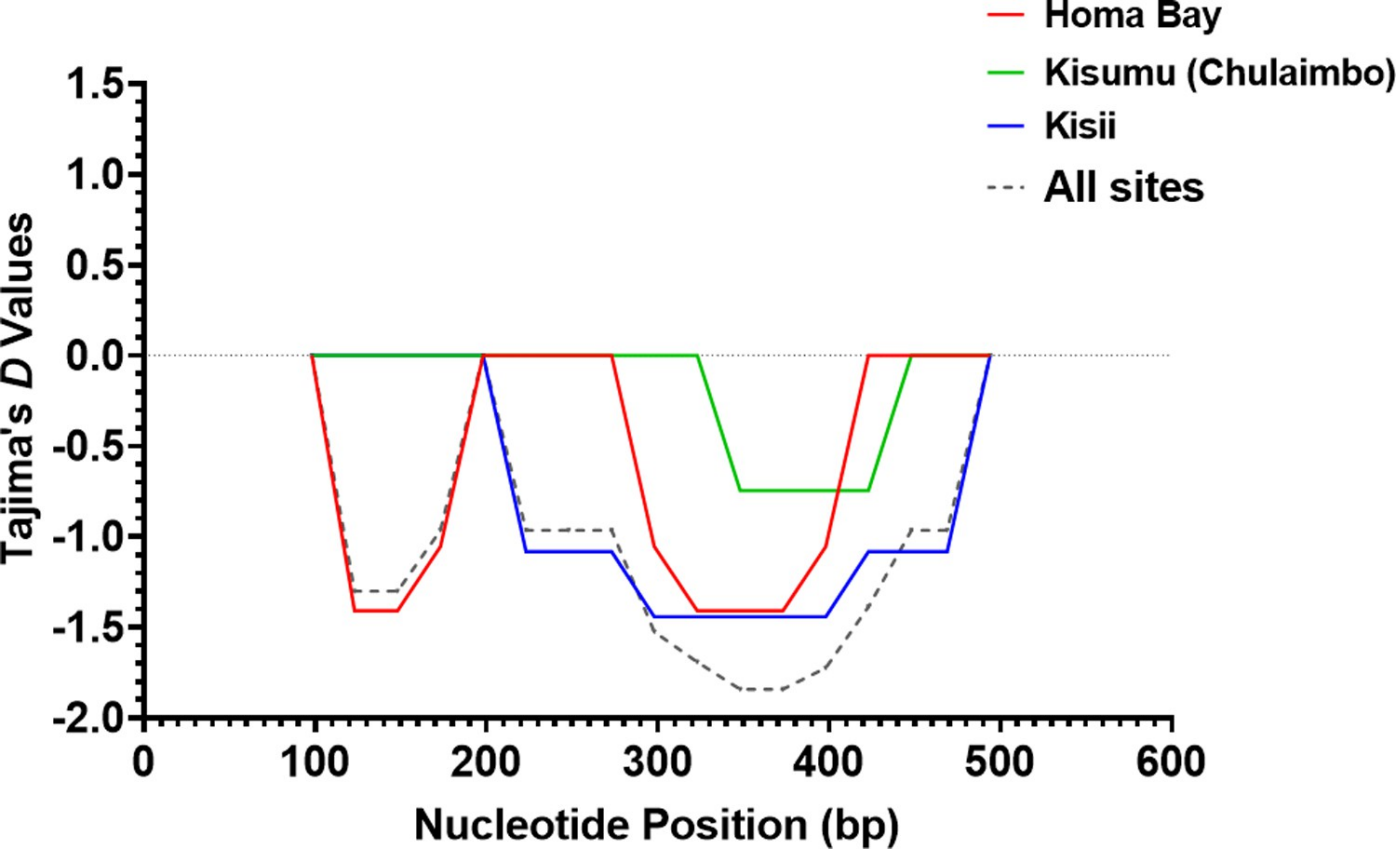
Sliding window plot of Tajima’s *D* values for *Pfs25* gene in western Kenya. The X-axis displays the nucleotide position (Window midpoint) whereas the Tajima’s *D* values are represented on the Y-axis. The red curve represents computed Tajima’s *D* value for Pfs25 sequences from *P*. *falciparum* circulating in Homa Bay, the green colour is for Chulaimbo, the blue colour is for Kisii whereas the black dotted colour represents the population from the three study sites. The middle horizontal dotted line (intersecting the Y-axis at 0.0) represents a standard neutral model where the Tajima’s *D* value is equal to zero. Positive deviation from the grey dotted line signifies balancing selection whereas negative deviation represents purifying selection.

The computed ratio of nonsynonymous and synonymous substitutions (d_N_/d_S_) for all Pfs25 sequences from western Kenya was 5.37 (**Table 1**). The d_N_/d_S_ ratio was not only greater than 1 across all sequences but also in each of the three study sites. Neutrality test results (Tajima’s *D*) for all the Pfs25 sequences were negative, thus signifying the presence of natural selection. The Tajima’s *D* value was -2.12 (p<0.05), Fu and Li’s D* test statistic, FLD*: -4.78 (p<0.02), Fu and Li’s F* test statistic, FLF*: -4.58 (p<0.02) and Fu’s *F*_*S*_ statistic: -10.91 (p<0.001). A similar trend was observed on Tajima’s *D*: -1.81 (p<0.05), and Fu’s *F*_*S*_: -4.42 (p = 0.011) values of Pfs25 sequences from Homa Bay. Other test results FLD*: -3.72 and FLF*: -3.50 were also significant (p<0.05). The selection pressure exacted its effect on loci existing between 98-148bp and 323-373bp in Pfs25 sequences from Homa Bay (**Fig 4**). These loci harbour the observed segregating sites in Homa Bay sequences. Among sequences from Chulaimbo parasites, Tajima’s *D* and Fu’s *F*_*S*_ were -0.73 and 0.8, respectively. All these test results were not significant (p>0.05), with FLD* and FLF* values of 0.77 and 0.37 respectively. Deviation from the standard neutral model among the Chulaimbo samples was observed on loci existing between 348-423bp where segregating sites were observed (**Fig 4**). Sequences from Kisii had significant (p<0.05) Tajima’s *D* (-1.84) and Fu’s *F*_*S*_ (-3.9) values. The FLD* and FLF* results were -3.54 (p<0.02) and -3.52 (p<0.02) respectively. Purging selection on loci blocks ranging from 223-469bp was observed for these sequences (**Fig 4**).

To further assess the presence or history of drivers shaping allelic patterns within the four domains of the Pfs25 antigen, we assessed a pair of loci that were under LD as well as having a history of recombination events (Rm). Three pairs of loci (123, 372), (364, 428) and (390, 394) within polymorphic Pfs25 sequences were observed to be under LD (**Table 3**). The three loci pairs had highly significant (P<0.001) LD values. The presence of significant LD values is an indicator of inbreeding as a driver for the observed sequence changes in each study site. None of mutated Pfs25 sequences from western Kenya had a history of recombination.

## Discussion

This study revealed novel polymorphic sites across the four immuno-dominant domains of the Pfs25 antigen. Most of these sites were dimorphic sites and involved Pfs25 D3. They resulted in 9 haplotypes that were unique (“private”) not only to Homa Bay, Kisumu or Kisii highlands but also to western Kenya. The *Pfs25* gene exhibited low nucleotide diversity and a high conservation index. Natural selection was identified to be the leading cause of these mutations with purifying selection purging on specific loci blocks. Additionally, inbreeding was established as an alternative driver for linked polymorphic loci in analysed sequences from each study site. There was high level of interbreeding in *P*. *falciparum* population from Homa Bay, Chulaimbo and Kisii zones.

Generally, low nucleotide diversity was observed in Pfs25 sequences from parasites population in western Kenya compared to Asia (0.11×10^−2^), Thailand (0.09×10^−2^), Cambodia (0.12×10^−2^), India (0.31×10^−2^), Brazil (0.18×10^−2^) and Vietnam (0.26×10^−2^) [49]. Chulaimbo sequences had high nucleotide variation compared to Homa Bay and Kisii. The observed variation between the two malaria-endemic sites and a close similarity between Homa Bay and Kisii implies that ongoing indoor residual spraying targeting malaria vectors within Homa Bay [50] may be directly or indirectly reducing the numbers of parasite clones or genetic diversity. Despite having high number of haplotypes across western Kenya, their observed number was within the set limit of the total number of segregating plus one (S+1), thus implying the population is under selective sweep or growth [48, 51]. Apart from the dimorphic site 428 (codon V143) which had previously been reported in Pfs25 sequences from Cambodia [31, 49], India [30, 31, 49] and now in Kisii highland, the remaining nine sites were novel. The amino acid change observed in this codon (V143G), however, did not correspond to documented V143A mutation following differences in base substitution at locus 428 (T/G as opposed to T/C) of the gene in one of the sequences from the Kisii area. None of the sequences from western Kenya had G131A mutations that had been previously observed in the parasite population from India and Cambodia [31]. However, three sequences from Chulaimbo study sites had mutations at a nearby codon V132I, and was observed at a frequency of 7.7%. Compared to its analogue in *Plasmodium vivax*, two mutated codons T130 and V132 identified in Pfs25 had been described in Pv25 in Asia [52, 53].

The majority of the polymorphic sites we discovered were in the *Pfs25* D3 region. This domain had previously been described to be highly immunogenic compared to others, and is also known for harboring binding sites of 1D2 and 4B7 human monoclonal antibodies used in standard membrane feeding assay studies of *P*. *falciparum* mosquito infectivity [14, 25, 54]. Among mutated codons in D3, codon change C110C and C115W targeted two of the 21 cysteine residues that are known to be highly conserved and function in maintaining the folding pattern of the Pfs25 polypeptide [14, 31, 55, 56]. The presence of such silent and nonsynonymous mutation points to the possibility of a weak or strong selection pressure acting on nearby codons within the Pfs25 antigen [57]. If pronounced, such mutation may interfere with the folding pattern of the Pfs25 antigen, and thereby impact interaction between host antibodies elicited by Pfs25 TBV and modify interactions with other B-cell epitopes as observed with other proteins [58] and cysteine residues in the D2 region of Pfs47 [58].

As opposed to previous reports describing the absence of selective pressure acting on the *Pfs25* gene [28], our studies suggest that natural selection may play a role in shaping allelic diversity of the *Pfs25* in western Kenya. Specifically, purifying selection was suggested based on computation of the ratio of nonsynonymous and synonymous substitutions [43–46]. This was evident across all mutated codon since they displayed a significant Tajima’s *D* value. The loci pairs under LD not only reaffirmed the history of selection but also confirmed inbreeding as another factor in the spread and sustenance of such mutations [59]. We speculate that selection pressure may be arising from unknown factor in protease-rich midgut of female Anopheline vectors.

## Conclusion

The *Pfs25* gene from the malaria-endemic and epidemic-prone region of western Kenya revealed varying levels of genetic diversity and novel haplotypes. Purifying selection and inbreeding were predicted as the cause and drivers for the observed variations. The low level of nucleotide diversity is an indicator that TBV based on Pfs25 sequences is less likely to be affected by antigenic variations. However, this study recommends continued monitoring of the *Pfs25* gene from different malaria-prone regions including areas where clinical trials have been conducted. This will not only aid in unravelling new polymorphic sites that could have an effect on antibody binding especially on immunogenic D3 but also monitor the durability of the *Pfs25* gene as a potential TBV.

## Supporting information

S1 Fig Sequence chromatograms for mutated loci within *Pfs25* gene from *Plasmodium falciparum* in western Kenya. The sequence chromatograms are aligned to the reference sequence (PF3D7_1031000). The vertical red arrows indicate the position of the SNPs. The asterisk (*) represents samples with mixed haplotype infections. The sequences are also deposited in GenBank with accession numbers MT212735-MT212808 and MT225462-MT225527.
(PDF)

S1 File Raw sequencing chromatograms. (RAR)

S1 Data (FAS)

S2 Data (FAS)

S3 Data (FAS)
