# Supplementary material for: Signatures of selection and drivers for novel mutation on transmission-blocking vaccine candidate Pfs25 gene in western Kenya
Source: PLoS One. 2022 Apr 7;17(4):e0266394. doi: 10.1371/journal.pone.0266394 (PMC8989228; doi:10.1371/journal.pone.0266394)
Supplement: S1 Fig — The sequence chromatograms are aligned to the reference sequence (PF3D7_1031000). The vertical red arrows indicate the position of the SNPs. The asterisk (*) represents samples with mixed haplotype infections. The sequences are also deposited in GenBank with accession numbers MT212735-MT212808 and MT225462-MT225527. (PDF) [file pone.0266394.s001.pdf]

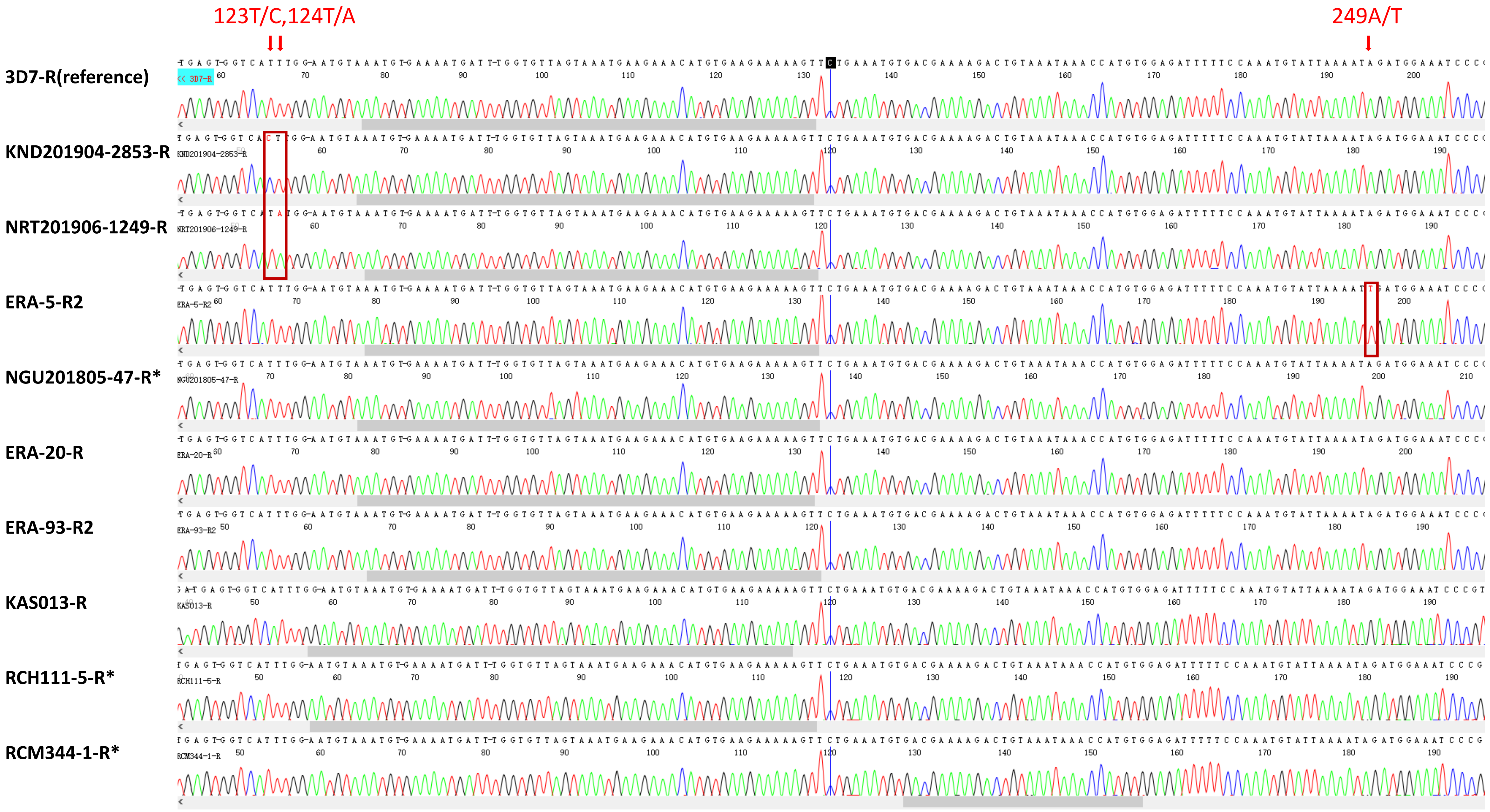

123T/C,124T/A
249A/T
3D7-R(reference)
KND201904-2853-R
NRT201906-1249-R
ERA-5-R2
NGU201805-47-R*
ERA-20-R
ERA-93-R2
KAS013-R
RCH111-5-R*
RCM344-1-R*

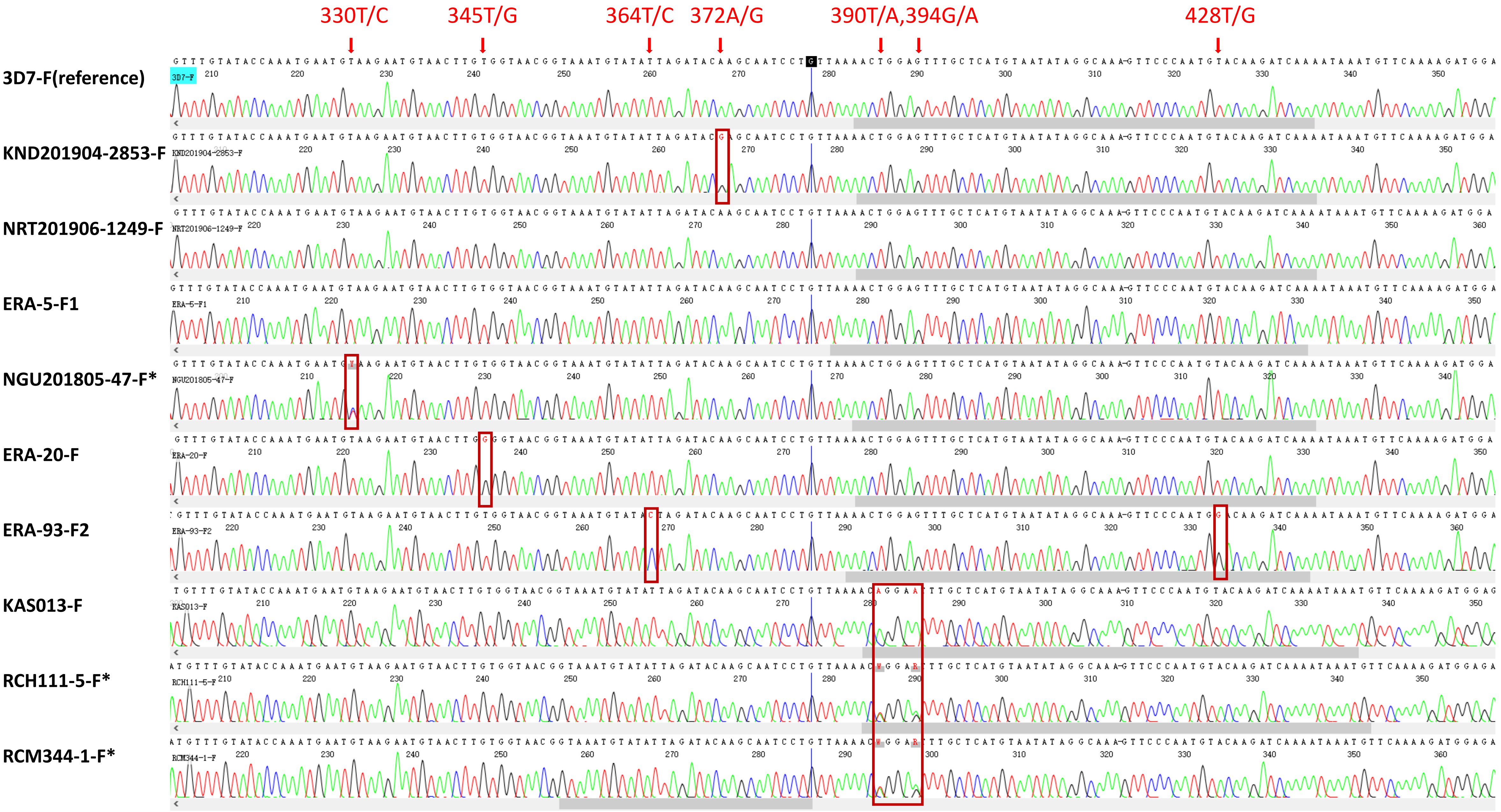
330T/C
345T/G
364T/C
372A/G
390T/A,394G/A
428T/G
3D7-F(reference)
KND201904-2853-F
NRT201906-1249-F
ERA-5-F1
NGU201805-47-F*
ERA-20-F
ERA-93-F2
KAS013-F
RCH111-5-F*
RCM344-1-F*
